# Drying alters the phenolic constituents, antioxidant properties, α‐amylase, and α‐glucosidase inhibitory properties of Moringa (*Moringa oleifera*) leaf

**DOI:** 10.1002/fsn3.770

**Published:** 2018-10-10

**Authors:** Adedayo O. Ademiluyi, Olubukola H. Aladeselu, Ganiyu Oboh, Aline A. Boligon

**Affiliations:** ^1^ Functional Foods and Nutraceuticals Unit Department of Biochemistry Federal University of Technology Akure Nigeria; ^2^ Postgraduate Programme in Pharmaceutical Sciences Universidade Federal de Santa Maria Santa Maria RS Brazil

**Keywords:** α‐amylase, α‐glucosidase, antioxidant properties, drying, *Moringa oleifera* leaf, phenolics

## Abstract

*Moringa oleifera* leaf is a popular green leafy vegetable which has found its usefulness in the preparation of traditional stews and soups. Like most green leafy vegetable which are not around year‐round, the leaf is usually dried and pulverized for storage and easier handling, and despite the popularity of this processing technique, there is dearth of information on how drying affects the health‐promoting properties of the leaves. Hence, this study sought to investigate the effect of some drying methods (freeze‐drying, sun, air and oven drying) on the phytoconstituents, antioxidant properties, and biological activities of moringa leaf. This study revealed that drying methods significantly altered the phytoconstituents (phenolics, flavonoids, vitamin C, tannin, saponin, phytate, oxalate, alkaloid, cardenolides, and cardiac glycosides), antioxidant capacities (reducing power, Fe^2+^chelating, ABTS ^•+^, DPPH, and ^•^ OH scavenging abilities), and enzyme inhibitory (α‐amylase and α‐glucosidase) effects of the leaf, with freeze‐drying being the most promising method for preserving the nutraceutical properties of moringa leaf. However, for practical application, the order of preference of the drying methods which ensures adequate retention of phytoconstituents and possibly biological activities of the leaf as observed in this study is freeze‐drying > air drying > sun drying > oven drying, in the order of decreasing magnitude.

## 1 INTRODUCTION

*Moringa oleifera* is a pan‐tropical plant having small‐ or medium‐sized perennial softwood tree with timber of low quality. It is the best known and most widely cultivated species of a monogeneric genus plant family of *Moringaceae*. This plant is native to sub‐Himalayan regions of northern India and has been planted around the world and naturalized in many locales (Martin, 2013). In Nigeria, it is known by many native names such as “zogeli” in Hausa, “okwe oyibo” in Igbo, “ewe ile,” “igi iyaanu,” or “ewe igbale” in Yoruba and “dogalla” in Taroh (Fahey, 2005; Saalu et al., 2011). It is considered one of the world's most useful trees because almost every part of the tree has some nutritional, medicinal, and other beneficial properties (Luqman, Srivastava, Kumar, Maurya, & Chanda, 2012). The medicinal properties have been attributed to phytochemical compositions of its various parts: the roots, bark, leaf, flowers, fruits, and seeds (Anwar, Latif, Ashraf, & Gilani, 2007; Kumar, Mishra, Ghosh, & Panda, 2010).

*Moringa oleifera* leaf belongs to the family of dark green leafy vegetables, which are particularly rich in nutrients. The leaves of *Moringa* are particularly good sources of proteins, calcium, iron, β‐carotene (converted to vitamin A in the human body), vitamin C, and vitamin E (Zaku, Emmanuel, Tukur, & Kabir, 2015). The most utilized component of Moringa is its leaf, and like most green leafy vegetables, the leaves are usually processed fresh or dried and kept for future usage. Green leafy vegetables are usually processed into soups and stews in many communities in Nigeria where they accompany a traditional starchy meal. “Efo‐riro” is a rich vegetable stew that is native to the Yoruba people of Western Nigeria. This particular stew involves the use of many different green leafy vegetables in its preparation and often times, moringa leaf is employed. The green leaves of *Moringa* are harvested and carefully washed to remove dirt. The leaf is blanched before incorporation into the stew or could be included directly without blanching. Furthermore, *Moringa* leaves are added to other vegetable soups like “Okra,” “Egusi,” “Ugwu,” and “Spinach” (Babayeju et al., 2014). In other parts of Nigeria, the young leaf is commonly cooked and eaten like spinach or used to make other soups and salads. Furthermore, the leaf is often consumed raw, cooked or dried and ground into fine powder which could be added to almost any food such as pap (Ogi) and other cereal gruels as nutrient supplement (Zaku et al., 2015).

*Moringa* have found usefulness in the folk medicine where the infusions, decoctions, and concoctions of various parts of this plant are used in the treatment of several ailments such as cardiac and circulatory stimulants; possesses antitumor, antipyretic, antiepileptic, anti‐inflammatory, antiulcer, antispasmodic, diuretic, antihypertensive, cholesterol lowering, antioxidant, antidiabetic, hepatoprotective, antibacterial, and antifungal activities (Anwar et al., 2007). And these health‐promoting effects have been attributed to its constituent phytochemicals such as zeatin, quercetin, β‐sitosterol, caffeoylquinic acid, and kaempferol (Anwar et al., 2007;. Furthermore, evidences are also pointing to antioxidant activity as one of the main mechanism of action underlying the medicinal properties of moringa leaf (Pari & Kumar, 2002).

Moringa leaf, like most green leafy vegetables in Nigeria, is not around year‐round; hence, they are usually processed dry using several local means such as sun drying, drying under shade, and oven drying. These dried leaves are pulverized and applied directly to soups as thickener and to several other food preparations. In an unfortunate way, despite the popularity of these practices which is mainly to extend the shelf‐life as well as the handling of this green leafy vegetable, there is dearth of information on the effect of these various drying methods on the phytoconstituents, nutritional and medicinal values of *M. oleifera* leaf. Hence, this study sought to investigate the effect of some drying methods (air‐, oven‐, sun‐, and freeze‐drying) on some phytoconstituents of moringa leaf and to ascertain how this affects its antioxidant properties and inhibitory effects on α‐amylase and α‐glucosidase activities in vitro.

## 2 MATERIALS AND METHODS

### 2.1 Plant material

Fresh Moringa (*M. oleifera*) leaf was obtained from a local Moringa plantation in Akure metropolis, Nigeria and authenticated at the Department of Crop, Soil and Pest Management, Federal University of Technology, Akure, Nigeria.

### 2.2 Animals

Adult wistar strain albino rats weighing 220–260 g were purchased from the Central Animal House of University of Ibadan and were maintained in wire mesh cages and fed with commercial rat chow and water ad libitum. The animals were acclimatized under this condition for 2 weeks prior to the study.

### 2.3 Chemicals and reagents

Folin‐Ciocalteu's reagent, thiobarbituric acid, 1,10‐phenanthroline, deoxyribose, gallic acid, trichloroacetic acid (TCA), dinitrophenyl hydrazine (DNPH), 1,1‐diphenyl‐2 picrylhydrazyl (DPPH) radical, 2,2‐azino‐bis(3‐ethyl‐benzothia‐zoline‐6‐sulfonic acid) (ABTS), catechin, epicatechin, quercetin, quercitrin, isoquercitrin, rutin, kaempferol, α‐amylase and α‐glucosidase were sourced from Sigma‐Aldrich, Inc. (St Louis, MO). Methanol, formic acid, gallic acid, chlorogenic acid, caffeic acid and ellagic acids were purchased from Merck (Darmstadt, Germany). All other chemicals and reagents used were of analytical grade and glass‐distilled water was used.

### 2.4 Sample preparation

The leaves were washed under running tap to remove dirt and drained in a plastic sieve. Thereafter, a portion of the leaves was freeze‐dried, another portion was sun dried, the third portion was air‐dried at room temperature and the last portion was oven dried (40°C). All the drying was carried out to constant weight. The dried samples were then pulverized and kept in air‐tight containers prior to analysis.

### 2.5 Aqueous extract preparation

One gram each of the powdered samples was weighed and extracted in 100 ml distilled water for 24 hr on an orbital shaker. The extract was further filtered using Whatman filter paper (No. 1) and the filtrate obtained was centrifuged at 1,000 × *g* for 10 min. Thereafter, the supernatant obtained was dried under vacuum using a rotatory evaporator and dried extract obtained was kept at −4°C for subsequent analysis (Oboh, Puntel, & Rocha, 2007). The percentage yield of the extracts were 44.2%, 45%, 43.6% and 43.7% for freeze‐dried, oven dried, air‐dried and sun dried samples, respectively.

### 2.6 Total phenol determination

The total phenol content was determined according to the method of Singleton, Orthofer, and Lamuela‐Raventos (1999). Appropriate dilutions of the extracts were oxidized with 2.5 ml 10% Folin‐Ciocalteu's reagent (v/v) and neutralized by 2.0 ml of 7.5% sodium carbonate. The reaction mixture was incubated for 40 min at 45°C and the absorbance was measured at 765 nm in the spectrophotometer. The total phenol content was subsequently calculated as gallic acid equivalent.

### 2.7 Total flavonoid determination

The total flavonoid content was determined using the method of Meda, Lamien, Romito, Millogo, and Nacoulma (2005). In brief, 0.5 ml of appropriately diluted sample was mixed with 0.5 ml methanol, 50 μl 10% AlCl_3_, 50 μl 1 M Potassium acetate and 1.4 ml water, and allowed to incubate at room temperature for 30 min. The absorbance of the reaction mixture was measured at 415 nm in the spectrophotometer and the total flavonoid content was subsequently calculated as quercetin equivalent.

### 2.8 Vitamin C content determination

Vitamin C content of the extracts was determined using the method of Benderitter, Maupoil, VeBriot, and Rochette (1998). In brief, 75 μl DNPH (2 g DNPH, 230 mg thiourea and 270 mg CuSO_4_.5H_2_O in 100 ml of 5 M H_2_SO_4_) were added to 500 μl reaction mixture (300 μl of appropriate dilution of the extracts with 100 μl 13.3% TCA and water). This was subsequently incubated for 3 hr at 37°C, then 0.5 ml of 65% H_2_SO_4_ (v/v) was added to the mixture and the absorbance was measured at 520 nm. The vitamin C content was subsequently calculated as ascorbic acid equivalent (AAE).

### 2.9 Tannin content determination

The tannin content was determined according to the method of Makkar and Goodchild (1996). In brief, 0.2 g of the sample was weighed into 50 ml sample bottle and 10 ml of 70% aqueous acetone was added and properly covered. The bottle was shaken for 2 hr at 30°C and the solution was centrifuged at 1,000 × *g* before the supernatant collected was stored in ice. Thereafter, 0.2 ml of the solution was mixed with 0.8 ml of distilled water and 0.5 ml of Folin‐ciocalteu reagent was added (the same amount of Folin reagent was added to 1 ml of 0.5 mg/ml standard tannic acid solution). Then, 2.5 ml of 20% Na_2_CO_3_ was added and the solutions vortexed and allowed to incubate for 40 min at room temperature. The absorbance of the reaction mixture was measured at 725 nm in a spectrophotometer. The tannin content was calculated as an equivalent of tannic acid.

### 2.10 Saponin content determination

The saponin content was determined using the method of Brunner (1994). In brief, 2 g of the powdered sample was weighed into a 250 ml beaker and 100 ml of isobutyl alcohol was added. The mixture was shaken for 5 hr and the mixture was filtered into 100 ml beaker containing 20 ml of 40% saturated magnesium carbonate (MgCO_3_) solution. The mixture obtained was also filtered to obtain a colorless clear solution. Then, 1 ml of the colorless solution was pipetted into 50 ml volumetric flask and 2 ml of 5% iron (III) chloride (FeCl_3_) solution was added and made up to the mark with distilled water (1 ml of 0.2 mg/ml saponin solution was used as control). The mixture was incubated at room temperature for 30 min and the absorbance was measured at 380 nm in a spectrophotometer. The saponin content was subsequently expressed as standard saponin equivalent.

### 2.11 Phytate content determination

The phytate content was determined according to Day and Underwood (1986). In brief, 4 g of the powdered sample was soaked in 100 ml of 2% HCl for 3 hr and filtered through No. 1 Whatman filter paper. Thereafter, 5 ml of 0.3% ammonium thiocyanate solution was added to 25 ml of the filtrate as indicator. In a subsequent way, 53.5 ml of distilled water was added to give it the proper acidity and this was titrated against iron (III) chloride solution that contained 1.95 mg of iron per milliliter until a brownish yellow color persisted for 5 min. The phytate content was subsequently calculated using the titer value obtained.

### 2.12 Oxalate content determination

The oxalate content was determined using the method of Day and Underwood (1986). In brief, 1 g of the powdered sample was soaked in 75 ml of 1.5 N H_2_SO_4_ for 1 hr and filtered through a No. 1 Whatman filter paper. Then, 25 ml of the filtrate was titrated hot (about 80–90°C) against 0.1 M KMnO_4_ until a pink color persisted for 15 s. The oxalate content was subsequently calculated using the titer value obtained.

### 2.13 Alkaloid content determination

The alkaloid content was determined according to the method of Harbone (1973). Five grams of the powdered sample was weighed and 200 ml of 10% acetic acid in ethanol was added, and the reaction mixture was incubated at room temperature for 4 min. This was filtered using No. 1 Whatman filter paper and the filtrate obtained was concentrated on a water bath to a quarter of the original volume. Concentrated NH_4_OH (20 mM) was added in drops to the concentrated filtrate until precipitation was completed. The whole solution was allowed to settle and the precipitate was collected, washed with dilute NH_4_OH (250 mM) and filtered. The recovered residue was weighed and quantified as the alkaloid.

### 2.14 Cardenolides content determination

The cardenolides content was determined according to the method of Dantas‐barros, Foulquier, Cosson, and Jacquin‐dubreuil (1993). In brief, 0.5 g of finely powdered sample was weighed and 50 ml of chloroform was added. Thereafter, 10 ml of 2% Na_2_CO_3_ was added to remove any free acid. The reaction mixture was later transferred into a 250 ml separating funnel and shaken thoroughly to allow the layers to separate with five drops of acetic anhydride being added. This mixture was filtered into 100 ml volumetric flask and made to the mark with chloroform. The absorbance was measured at 510 nm in a spectrophotometer. Standard cardenolide solution (10 mg/ml) was used as control and the cardenolide content was subsequently calculated as the equivalent of the standard solution.

### 2.15 Cardiac content determination

The cardiac glycoside content was determined according to Sofowora (1993). One gram of the powdered sample was weighed, 50 ml chloroform was added and vortexed for 1 hr. The reaction mixture was filtered, followed by the addition of 10 ml of pyridine and 2 ml of 29% sodium nitroprusside and the mixture was shaken thoroughly for 10 min. Thereafter, 3 ml of 20% NaOH was added in order to develop a brownish color and the absorbance was read at 510 nm in a spectrophotometer. Standard cardiac glycosides (Digitoxin) solution (5 mg/ml) was used as the control and the cardiac glycoside content was subsequently calculated as the equivalent of the standard solution.

### 2.16 Antioxidant assays

Free radical scavenging ability was determined by assessing the ability of the moringa leaf extracts to bleach stable DPPH radical as reported by Gyamfi, Yonamine, and Aniya (1999). To correct for the limitations of DPPH assay (color interference and sample solubility), the radical scavenging ability was further tested using ABTS. The assay principle is based on scavenging of ABTS^•+^ formed from treatment of ABTS with sodium persulfate. This radical cation (ABTS^•+^) is blue in color and absorbs light at 734 nm. The blue colored ABTS radical is converted back to its original colorless neutral form by antioxidant molecules and the extent of bleaching is measured as trolox equivalent antioxidant capacity (Re et al., 1999). The reducing property (FRAP) of the extracts was determined by assessing the ability of the extracts to reduce Fe^3+^ to Fe^2+^ in solution as described by Oyaizu (1986). The hydroxyl radical (^•^OH) scavenging assay was based on the ability of the extracts to scavenge/prevent ^•^OH production from Fe^2+^/H_2_O_2_‐induced decomposition of deoxyribose in solution. The Fe^2+^ chelating ability of the extracts was determined using a modified method of Minotti and Aust (1987) with a slight modification by Puntel, Nogueira, and Rocha (2005). Furthermore, the ability of the extracts to prevent both FeSO_4_ and sodium nitroprusside‐induced lipid peroxidation in rat's pancreas and liver homogenates was studied (Ohkawa, Ohishi, & Yagi, 1979).

### 2.17 α‐Amylase inhibition assay

The α‐amylase inhibitory activity was determined as described by Worthington and Worthington (1993). Appropriate concentration of the extracts and 50 μl of 20 mM sodium phosphate buffer (pH 6.9 with 6 mM NaCl) containing pancreatic α‐amylase (EC 3.2.1.1) (0.5 mg/ml) were incubated at 25°C for 10 min. Then, 50 μl of 1% starch solution (prepared in the same buffer) was added and reaction mixture was incubated at 25°C for 10 min. Thereafter 200 μl of dinitrosalicylic acid (DNSA) was added and the reaction stopped by incubating in a boiling water bath for 5 min. This was later cooled to room temperature and diluted with 2 ml of distilled water, and absorbance measured at 540 nm. The α‐amylase inhibitory activity was expressed as percentage (%) inhibition.

### 2.18 α‐Glucosidase inhibition assay

The α‐glucosidase inhibitory activity was determined as described by Apostolidis, Kwon, and Shetty (2007). Appropriate concentration of the extracts and 100 μl of α‐glucosidase (EC 3.2.1.20) solution in 100 mM phosphate buffer (pH 6.9) were incubated at 25°C for 10 min. Thereafter, 50 μl of 5 mM *p*‐nitrophenyl‐α‐d‐glucopyranoside solution (in the same buffer) was added. The mixture was incubated at 25°C for 5 min, before reading the absorbance at 405 nm. The α‐glucosidase inhibitory activity was expressed as percentage (%) inhibition.

### 2.19 HPLC‐DAD analysis

High‐performance liquid chromatography (HPLC‐DAD) was performed with a Shimadzu Prominence Auto Sampler (SIL‐20A) HPLC system (Shimadzu, Kyoto, Japan) and equipped with Shimadzu LC‐20AT reciprocating pumps connected to a DGU 20A5 degasser with a CBM 20A integrator, SPD‐M20A diode array detector and LC solution 1.22 SP1 software. The quantification of phenolic compounds in the differently dried moringa leaf was carried out using the method described by Waczuk et al. (2015) with slight modifications. Reverse phase chromatographic analyses were carried out under gradient conditions using C_18_ column (4.6 mm × 150 mm) packed with 5 μm diameter particles; the mobile phases A and B were Milli‐Q water, acidified to pH 2.0 with 1% of phosphoric acid and methanol respectively, solvent gradient was used as follows: 0–10 min, 5% B; 10–25 min, 15% B; 25–40 min, 30%; 40–55 min 50% B; 50–65 min 70% B; 65–80 min, 100% B, respectively. The flow rate was 0.6 ml/min and the injection volume was 50 μl. Quantifications were carried out by integration of the peaks using the external standard method, at 254 nm for gallic and ellagic acids; 280 nm for catechin and epicatechin; 327 nm for chlorogenic and caffeic acids; and 366 for quercetin, kaempferol, and rutin.

### 2.20 Statistical analysis

The results of the three replicate experiments were pooled and expressed as mean ± standard deviation (*SD*). A one‐way analysis of variance was used to analyze the mean, and the post hoc treatment was performed using Duncan multiple test. Significance was accepted at *p* ≤ 0.05.

## 3 RESULTS

The effects of some drying methods on phytochemical composition of *M. oleifera* leaf revealed variations in their constituents. The results of phenolics, flavonoid, vitamin C, tannin, saponin, phytate, oxalate, alkaloid, cardenolides and cardiac glycosides content in the differently dried moringa leaf are presented in Table 1. The result revealed that freeze‐dried sample had significantly (*p* < 0.05) the highest composition of phytochemicals while oven‐dried sample had the lowest. However, irrespective of the drying method, phenolics, flavonoids, vitamin C, and phytate were the most abundant while tannin was the least abundant phytoconstituents in the moringa leaf.

**Table 1:** Effects of drying on the phytochemical constituents of *Moringa oleifera* leaf

Parameters	Freeze‐dried (mg/g)	Air‐dried (mg/g)	Sun dried (mg/g)	Oven dried (mg/g)
Phenolics	68.75 ± 0.00^d^	59.38 ± 0.42^c^	50.00 ± 0.00^ab^	46.88 ± 1.42^a^
Flavonoid	62.50 ± 0.89^d^	58.33 ± 0.00^cd^	45.83 ± 0.89^b^	25.00 ± 0.00^a^
Vitamin C	52.94 ± 0.31^d^	41.17 ± 0.31^c^	35.29 ± 0.63^bc^	23.53 ± 0.60^a^
Tannin	0.06 ± 0.03	0.05 ± 0.02	0.05 ± 0.03	0.05 ± 0.03
Phytate	70.26 ± 2.40^c^	89.82 ± 0.98^d^	60.98 ± 0.00^ab^	58.50 ± 1.42^a^
Saponin	16.36 ± 0.92^c^	16.36 ± 0.00^c^	10.91 ± 0.82^b^	7.27 ± 0.71^a^
Alkaloid	12.8 ± 1.71^c^	13.4 ± 0.00^c^	5.00 ± 0.92^a^	10.6 ± 2.41^b^
Oxalate	9.96 ± 0.84^c^	9.09 ± 0.72^c^	6.66 ± 0.00^a^	8.19 ± 0.60^b^
Cardenolides	13.68 ± 0.71^b^	11.72 ± 1.90^b^	12.53 ± 2.40^b^	8.17 ± 1.71^a^
Cardiac glycosides	17.36 ± 1.31^b^	16.72 ± 1.91^b^	14.79 ± 2.81^a^	14.79 ± 1.82^a^

*Note*

Values represent mean ± standard deviation of triplicate experiments. Superscripts with different alphabets along the same row are significantly (*p* < 0.05) different.

The effect of the various drying methods on the DPPH free radical scavenging ability of the moringa leaf was studied and the result is presented in Table 2. The result revealed that aqueous extracts scavenged DPPH radicals in a concentration‐dependent (0–330 mg/ml) pattern. However, drying altered the DPPH radical scavenging ability of the leaves as freeze‐dried sample had significantly (*p* < 0.05) the highest radical scavenging ability (251 mg/ml). The effect of the drying methods on the antioxidant capacity of the moringa leaf was further studied using a moderately stable nitrogen‐centered radical specie, ABTS^•+^; as presented in Table 2. The result also showed that the various drying methods significantly (*p* < 0.05) altered the antioxidant capacity of the moringa leaf in a manner similar to the DPPH radical scavenging ability. This is evident by the fact that freeze‐dried leaf with the highest DPPH scavenging ability also had the highest ABTS^•+^ scavenging ability (1.25 ± 0.05 mmol TEAC/g). Furthermore, the ferric reducing antioxidant property of the Moringa leaves was also studied and expressed as AAEs (Table 2). The freeze‐dried leaf had the highest (12.66 ± 0.46 mg AAE/g) reducing power while the oven‐dried leaf had the least (7.47 ± 0.46 mg AAE/g). The effect of the drying methods on hydroxyl (OH*) radical scavenging ability of the aqueous extracts of moringa leaf is also presented in Table 2. The result showed that freeze‐dried leaves had the highest ^•^OH scavenging ability (61.25 mg/ml) while oven‐dried leaves had the least (116.18 mg/ml**)**. Moreover, the effect of the drying methods on Fe^2+^ chelating ability of the moringa leaf was investigated and the result is presented in Table 2. The result also revealed that extracts of the differently dried Moringa leaves chelate Fe^2+^ in a concentration‐dependent (0–100 mg/ml) pattern. However, these drying methods significantly (*p* < 0.05) altered the Fe^2+^ chelating property of the leaves; with freeze‐dried leaf having the highest Fe^2+^ chelating ability (73.14 mg/ml**)** while oven‐dried leaf had the least (197.89 mg/ml**)**.

**Table 2:** Effects of drying on the DPPH and ^•^OH scavenging ability, ABTS^•+^ scavenging, and ferric reducing antioxidant properties (FRAP), Fe^2+^‐chelating, inhibition of lipid peroxidation, α‐amylase, and α‐glucosidase activities of *Moringa oleifera* leaf

Parameters	Freeze‐dried	Air‐dried	Sun dried	Oven dried
Antioxidant properties				
DPPH (mg/ml)^a^	251.42 ± 1.03^a^	275.88 ± 0.14^b^	337.91 ± 0.02^d^	310.56 ± 0.03^c^
ABTS^•+^ (mmol TEAC/g)	1.25 ± 0.05^b^	1.14 ± 0.11^ab^	1.02 ± 0.00^a^	0.98 ± 0.00^a^
FRAP (mg AAE/g)	12.66 ± 0.46^c^	11.04 ± 0.91^bc^	9.42 ± 0.46^**b**^	**7**.47 ± 0.46^a^
^•^OH (mg/ml)^a^	61.25 ± 0.03^a^	80.36 ± 0.14^b^	94.31 ± 0.28^c^	116.18 ± 1.41^d^
Fe^2+^ Chelation (mg/ml)^a^	73.14 ± 0.03^a^	85.39 ± 0.04^b^	142.75 ± 0.03^c^	197.89 ± 0.01^d^
IC_50_ for inhibition of Fe^2+^‐induced lipid peroxidation (mg/ml)				
Pancreas	26.14 ± 1.86^a^	31.92 ± 0.14^b^	32.52 ± 0.28^c^	35.09 ± 0.06^d^
Liver	32.03 ± 1.26^a^	35.43 ± 1.41^c^	34.04 ± 0.06^b^	38.92 ± 0.03^d^
IC_50_ for inhibition of sodium nitroprusside‐induced lipid peroxidation (mg/ml)				
Pancreas	37.41 ± 0.72^a^	38.51 ± 0.02^b^	44.26 ± 0.06^d^	42.53 ± 0.02^c^
Liver	36.20 ± 0.12^a^	39.53 ± 0.28^b^	40.68 ± 0.02^c^	42.23 ± 0.04^d^
IC_50_ for enzymes inhibition (mg/ml)				
α‐Amylase	64.29 ± 0.52^a^	73.47 ± 0.81^c^	69.90 ± 0.14^b^	81.82 ± 0.03^d^
α‐Glucosidase	38.12 ± 0.71^a^	42.52 ± 0.14^b^	46.16 ± 0.09^c^	51.27 ± 0.10^d^

*Notes*

Values represent mean ± standard deviation of triplicate experiments. Superscripts with different alphabets along the same row are significantly (*p* < 0.05) different.

a Represent the IC_50_ values (the amount of the extracts causing 50% antioxidant or enzyme inhibitory activity).

The results of lipid peroxidation presented in Table 2 as IC_50_ values showed that all the variously dried moringa leaf extracts inhibited MDA production in both the pancreas and liver in a concentration‐ (0–63 mg/ml) dependent manner. However, drying methods significantly (*p* < 0.05) altered this property. Also, the results as revealed in Table 2 (IC_50_) showed that all the extracts of the differently dried moringa leaf exhibited α‐amylase and α‐glucosidase inhibitory properties in a concentration‐ (0–80 mg/ml) dependent manner. Nevertheless, significant (*p* < 0.05) alteration in the inhibition of these enzymes was observed as affected by the different drying methods.

The result of some drying methods on phenolics and flavonoids composition of the extracts of dried moringa leaf is presented in Table 3. This revealed that gallic acid, catechin, epicatechin, chlorogenic acid, caffeic acid, ellagic acid, quercetin, rutin, and kaempferol were the predominant phenolics constituents of the moringa leaf. The result showed that there was variation in the phenolics and flavonoids content of the extracts of dried moringa leaf sample. However, gallic acid, chlorogenic acid, caffeic acid, and rutin were the most abundant in phenolics and flavonoids composition of the differently dried moringa leaf with freeze‐dried sample having the highest chlorogenic acid, caffeic acid and rutin constituents.

**Table 3:** Effects of drying on the phenolic constituents of *Moringa oleifera* leaf

Compounds	Freeze‐dried	Air‐dried	Sun dried	Oven dried	LOD	LOQ
	mg/g				μg/ml	
Gallic acid	43.19 ± 0.02^a^	58.35 ± 0.01^b^	41.06 ± 0.01^a^	60.11 ± 0.03^bc^	0.027	0.093
Catechin	6.08 ± 0.01^a^	7.13 ± 0.03^a^	5.98 ± 0.01^a^	29.76 ± 0.01^b^	0.009	0.034
Chlorogenic acid	79.53 ± 0.01^c^	63.19 ± 0.03^a^	62.35 ± 0.04^a^	65.83 ± 0.01^b^	0.011	0.037
Caffeic acid	78.91 ± 0.03^c^	62.81 ± 0.02^ab^	78.17 ± 0.03^c^	58.72 ± 0.02^a^	0.024	0.080
Ellagic acid	5.86 ± 0.01^a^	31.04 ± 0.01^c^	6.09 ± 0.01^a^	19.65 ± 0.02^b^	0.008	0.025
Epicatechin	43.37 ± 0.04^c^	27.76 ± 0.01^b^	18.63 ± 0.01^a^	28.95 ± 0.01^b^	0.019	0.063
Rutin	91.05 ± 0.01^c^	75.38 ± 0.04^b^	89.14 ± 0.02^c^	70.21 ± 0.03^a^	0.023	0.076
Quercetin	17.83 ± 0.01^a^	59.01 ± 0.01^b^	62.17 ± 0.03^c^	19.87 ± 0.03^a^	0.015	0.048
Kaempferol	43.90 ± 0.02^d^	40.11 ± 0.01^c^	9.58 ± 0.01^a^	19.65 ± 0.02^b^	0.021	0.069

*Note*

Values represent mean ± standard deviation of triplicate experiments. Means followed by different letters along the same row are significantly (*p* < 0.05) different.

## 4 DISCUSSION

Sun drying and air drying at room temperature are the most common practices used in many parts of the world to preserve vegetables for dry season consumption while freeze‐drying and oven drying are rarely used (Lyimo, Nyagwegwe, & Mukeni, 1991). However, these processing techniques may significantly affect the concentration and bioavailability of some essential constituents of the food. The phytochemical analysis of the moringa leaf not only showed the presence of phenolics, flavonoids, vitamin C, tannin, saponin, phytate, oxalate, alkaloid, cardenolides and cardiac glycosides but also revealed a variation in their concentration after being subjected to different drying processes. Furthermore, drying significantly (*p* < 0.05) altered the phytochemical constituents of the moringa leaf with the exception of tannin content which was not altered by drying. The presence of these phytochemicals in the moringa leaf may contribute to its medicinal value (Bakare, Magbagbeola, Akinwande, & Okunowo, 2010; Okwu & Morah, 2007).

Phenolics are one of the most effective antioxidant constituents of green leafy vegetables and studies have shown that the antioxidant properties of plant foods are directly proportional to their phenolic content (Chu, Sun, Wu, & Liu, 2002). Hence, the effect of the different drying methods on the phenolic contents of the moringa leaf was studied. The study revealed that the freeze‐dried sample had the highest phenolic content while oven‐dried sample had the least. This is consistent with earlier report that thermal treatments negatively impact the phenolic content of vegetables with a concomitant reduction in their antioxidant activity (Ismail, Marjan, & Foong, 2004). Hence, the observed decrease in the phenolic contents of the moringa leaf exposed to heat processes such as oven and sun drying could be due to heat‐induced degradation of phenolic compounds (Oboh, Akinyemi, Ademiluyi, & Adefegha, 2010). Furthermore, the flavonoid content of the differently dried moringa leaf followed same trend with the phenolics. The observed high flavonoid content of the freeze‐dried leaf may be due to the fact that some effective volatile compounds might have been destroyed during the other drying processes employed. It has been reported that heat treatment results in the degradation of flavonoids in vegetables (Mohd‐Zainol, Abdul‐Hamid, Abu‐Bakar, & Pak‐Dek, 2009). This might be responsible for the significant decrease (*p* < 0.05) observed in the flavonoid content of the oven‐dried leaf. Flavonoids have been reported as one of the most numerous and widely spread group of phenolics in higher plants (Carini, Adlini, Furlanetto, Stefani, & Facino, 2001; Schinella, Tournier, Prieto, Mordujovich de Buschiazzo, & Rios, 2002). Consumption of flavonoid‐rich foods has been suggested to be effective in reducing the risk of coronary heart diseases and other related diseases (Okwu & Omodamiro, 2005).

Ascorbic acid (vitamin C) has been described as an antioxidant found in plant foods that helps build up the body's defenses against free radicals (Ajiboye, 2009). It is a good reducing agent and exerts its antioxidant activities by electron donation (Oboh, 2008). Also, higher retention of vitamin C was observed in the freeze‐dried sample, this may be due to the fact that the leaves were not exposed to direct heat and air as vitamin C is rapidly oxidized on exposure to heat and air. This suggests that freeze‐drying has an edge over sun drying and oven drying in its preservation of the vitamin C content which could serve as a good dietary supplement for ascorbic acid. The vitamin C content of the moringa leaf was higher than that of some common green leafy vegetables consumed in Nigeria (Oboh, Akindahunsi, & Oshodi, 2002; Yadav & Sehgal, 1997). Tannins are phytochemical compounds of sufficiently high molecular weight containing sufficient hydroxyl and carboxyl groups which form effectively strong complexes with protein and other macromolecules. They are well‐known antioxidants in medicinal plants, foods, and fruits with multifunctional properties beneficial to human health. The tannin content was found to be very low and there was no significant (*p* < 0.05) difference observed (Table 1) in all the samples after treatment under the various drying methods. This may probably be due to little influence of drying methods on tannin content (Akanji, Ologhobo, Emiola, Adedeji, & Adedeji, 2003). Kumari and Jain (2012) reported that tannins are usually present in low amounts in plants. It has been reported that tannins have antioxidative and antidiabetic effects and this may be due to their binding ability with physiologically relevant protein and carbohydrates, which results in reduction in the bioavailability of carbohydrate and its digestive enzymes (i.e., α‐ amylase and α‐ glucosidase) (Kunyanga, Okoth, Imungi, & Vellingiri, 2011). In addition, they also inhibit insulin degradation and improve glucose utilization and may be relevant in the management of diabetes (Kumari & Jain, 2012). Furthermore, the tannin content of the moringa leaf falls below the reported critical value of 7.3–9.0 mg/g which could elicit toxicity (Aletor, 1993).

Phytate is the primary storage form of phosphorus and inositols in seeds, grains, a few tubers and fruits. The air‐dried sample had the highest phytate concentration while oven‐dried sample had the least. This may be attributed to the fact that heat reduces phytate content in plant foods as a result of leaching which might affect their extractability. Oboh et al. (2002) reported that food processing techniques such as thermal processing reduce phytate content in plant foods. It has also been reported that phytate inhibits α‐amylase and this may prove useful in the management of hyperglycemia (Lee, Park, Chung, & Cho, 2006). Furthermore, the interaction of phytate with starch and divalent metals has been reported to result in low glycemic index and also reduces the participation of iron in metal‐induced oxidative stress related with diabetes (Schlemmer, Frolich, Prieto, & Grases, 2009). Saponins are plant glycosides that form soapy lathers when mixed or agitated with water, used in detergents, foaming agents, and emulsifiers (Attia‐Ismail, 2015). These groups of compounds are extremely diverse in biological activities which are mostly related to their structure and sources. Saponins significantly affect feed intake and growth in animals (Das et al., 2012) which might be related to its effect on protein digestibility (Potter, Jimenez‐Flores, Pollack, Lones, & Berber‐Jimenez, 1993). The values obtained for saponin content were higher than those reported by Mbah, Ogbusu, and Eme (2012). However, as observed from this study, both freeze‐drying and air drying did not alter the saponin content of the leaves, while both sun drying and oven drying significantly (*p* < 0.05) reduced the saponin content. This observed reduction in the saponin content may be attributed to heat‐induced degeneration involved in both drying processes. In like manner, this finding was consistent with the findings of Mbah et al. (2012) where both sun drying and oven drying significantly reduced the saponin contents of Moringa leaves.

Alkaloids are the largest naturally occurring secondary substances with one or more nitrogen atom(s) in a heterocyclic ring. They are widely employed in medicine because of their physiological activities on humans and other animals (Imohiosen, Gurama, & Lamidi, 2014). The value obtained for alkaloid in this study was higher than the 4.28 mg/g reported by Oladeji, Taiwo, Gbadamosi, Oladeji, and Ishola (2017) for *M. oleifera* leaf. This content of alkaloid could be responsible for the slight bitter taste observed in the leaf. However, this study shows that sun drying and oven drying reduced the alkaloid content of the leaf. Oxalate occurs naturally in plants and it is synthesized via incomplete oxidation of carbohydrate. The sun dried leaf sample had the lowest oxalate content while the freeze‐dried sample had the highest content. The observed high oxalate content in the freeze‐dried compared to other samples processed differently, could be due to the effect of heat on oxalate stability.

In general, significant (*p* < 0.05) decrease in phytoconstituents was observed for both sun and oven‐dried samples; this may be attributed to the effect of UV‐radiation, speed and humidity of the wind, as well as high temperatures involved in sun drying and moist heat in oven‐drying process. This is consistent with earlier findings of Shilpi, Sabrina, and Nissreen (2011) where a decrease in phytochemical content of edible Irish brown seaweed as temperature increase (upon drying) was reported. Hence, the variations observed in the phytochemical composition of the moringa leaf might be due to the different heat‐induced chemical modifications that took place during the drying process.

DPPH is a relatively more stable nitrogen‐centered free radical donor that accepts an electron or hydrogen upon reduction to become a stable diamagnetic molecule. A compound with tendency to perform this reaction is an important factor in evaluating antioxidant activity and therefore a free radical scavenger (Hu, Zhang, & Kitts, 2000). The trend obtained in this study agreed with the phytochemical distribution in the moringa leaf and it is consistent with earlier studies (Amic & Davidovic‐Amic, 2003; Chu et al., 2002). Moreover, the ability of the aqueous extracts of the leaf to reduce Fe^3+^ to Fe^2+^ was also studied. This study revealed that drying process significantly (*p* < 0.05) affected the reducing power of the moringa leaf. The pattern of alteration by the drying method is also similar to that of the ABTS^•+^ scavenging ability with freeze‐dried leaf having the highest reducing power and the oven‐dried leaf having the least. The observed high reducing power of the freeze‐dried leaf as compared with others may be due to its high phytoconstituents. Reducing agents are potent terminators of oxidation process, and two mechanisms available for this property include (a) electron transfer and (b) hydrogen atom transfer (Rice‐Evans, Miller, & Paganga, 1996). Likewise, the ability of the drying methods to alter the inhibition of hydroxyl radical (^•^OH) produced from the degradation of deoxyribose through the Fenton reaction by moringa leaf extracts was studied. This study revealed that drying significantly (*p* < 0.05) altered the ability of moringa leaf to prevent ^•^OH production in vitro. The freeze‐dried leaves had the highest ^•^OH scavenging ability while oven‐dried leaves had the least. The trend in this result agreed with the effect of various drying methods on the phytochemical contents and antioxidant activities (ABTS^•+^, DPPH and ^•^OH scavenging abilities and reducing power) of the moringa leaf earlier discussed. Iron (Fe) is necessary for a lot of biochemical and physiological functions in biological systems; however, free cytosolic and mitochondria Fe can induce oxidative damage through its participation in reactions leading to ROS production (Oboh & Rocha, 2007); this gives credence to the use of iron chelators as therapy for iron overload. The in vitro total antioxidant analysis revealed that highest antioxidant activity is found in freeze‐dried moringa leaf with least in the oven‐dried leaf. This agreed with the phytochemical distribution in the leaf and is consistent with earlier studies where strong correlation existed between phytochemical contents and antioxidant properties of some plant foods (Chu et al., 2002; Hu et al., 2000).

Furthermore, peroxidation of membrane lipids and other macromolecules to give rise to reactive aldehydes and other electrophiles leading to cell and tissue damage has been known as hallmark of oxidative stress; hence determination and quantification of the reactive aldehydes (such as malondialdehyde, MDA) and other electrophiles formed has been used as a measure of oxidative damage in a biological system and also the extent of lipid peroxidation. Ability of vegetable and plant extracts to inhibit the MDA production has been used as an indication of their antioxidant power in a biological system. Hence, it is desirable to investigate the effect of the various drying methods on the ability of the dried moringa leaf extracts to prevent both Fe^2+^ and sodium nitroprusside‐induced MDA production in rat pancreas and liver homogenates in vitro. The pattern of alteration of lipid peroxidation is similar to the antioxidant test carried out in that, freeze‐dried moringa leaf exhibited the highest inhibitory effect and oven‐dried leaf had the least. This also agreed with the result obtained for phytoconstituents as affected by the drying methods. Hence, it is safe to suggest that alteration in the phytoconstituents as affected by drying methods could be principal modulator of the antioxidant and possibly the biological properties of moringa leaf; as previous studies have come to conclusion that antioxidant properties of plant food are directly proportional to its phytoconstituents.

Pancreatic α‐amylase catalyzes the breakdown of starch (polysaccharide) into disaccharides and oligosaccharides while intestinal α‐glucosidase is responsible for the breakdown of disaccharides to glucose which is absorbed from small intestine into the blood circulation. Inhibition of these enzymes has been adopted as dietary means for the control of postprandial hyperglycemia in diabetics. Reports have shown the use of moringa leaf in the management of diabetes (Jaiswal, Rai, Kumar, Mehta, & Watal, 2009), with its effect linked to the inhibition of carbohydrate hydrolyzing enzymes such α‐amylase and α‐glucosidase (Toma, Makonnen, Mekonnen, Debella, & Addisakwattana, 2014). However, there is dearth of information on how drying affects these properties. Hence, the effect of drying methods on the α‐amylase and α‐glucosidase activities was investigated. The pattern of alteration of α‐amylase and α‐glucosidase inhibitory properties is similar to the ones observed for the antioxidant studies, this suggests that drying‐induced alteration to the phytoconstituents and this may also be responsible for the observed enzyme inhibition pattern. Phytochemicals have been shown to be responsible for the antidiabetic effect of many medicinal and plant foods through their interaction with critical enzymes involved in carbohydrate metabolisms (Ademiluyi & Oboh, 2012). This function has been attributed majorly to the phenolic compounds (Toma et al., 2014) and recently, to some alkaloids (Tiong et al., 2013). Hence, factors that negatively affect the amounts and bioavailability of these compounds may significantly impact their antidiabetic properties.

**Figure 1:**
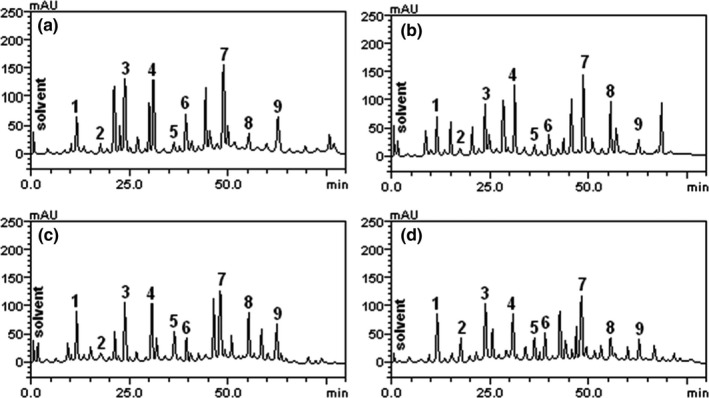
Representative high‐performance liquid chromatography profile of (a) freeze‐dried (b) sun dried (c) air‐dried and (d) oven dried *Moringa oleifera* leaf. Gallic acid (peak 1), catechin (peak 2), chlorogenic acid (peak 3), caffeic acid (peak 4), ellagic acid (peak 5), epicatechin (peak 6), rutin (peak 7), quercetin (peak 8), and kaempferol (peak 9)

Moringa leaf like many other green leafy vegetables contains phytochemicals such as phenolics which have been attributed for their health‐promoting effect. However, reports are on the increase about the effect of processing on the content and bioavailability of this important class of plant phytochemicals in green leafy vegetables. Hence, to ascertain the effect of the various drying methods on the phenolic constituents of the treated moringa leaf, HPLC‐DAD was employed. However, the effect of the drying methods on the phenolic constituents of the leaf did not totally agree with the antioxidant and enzyme inhibitory effect as the observed earlier trend was not totally followed. However, the phenolics and flavonoids constituents were significantly altered by the different drying methods used in this study. Chlorogenic acid, gallic acids, caffeic acid and rutin had the highest peaks; these were the most abundant phenolics and flavonoids composition in the dried moringa leaf. The freeze‐dried sample had the highest chlorogenic acid, caffeic acid and rutin constituents; this is consistent with earlier findings whereby freeze‐dried moringa leaf had highest phenolics content. The highest phenolics composition (Figure 1a) in chlorogenic acid, caffeic acid and rutin compounds may contribute to the highest α‐amylase and α‐glucosidase inhibition, prevention of lipid peroxidation and antioxidant properties obtained in the freeze‐dried moringa leaf. Chlorogenic acids are formed by the esterification of cinnamic acids, such as caffeic, ferulic, and p‐coumaric acids, with quinic acid. The consumption of chlorogenic acids has a lot of health benefits (Oboh, Agunloye, Akinyemi, Ademiluyi, & Adefegha, 2013). Gallic acid is a trihydroxybenzoic acid found both in free form and in esterified form as part of hydrolyzable tannins (gallotannins and ellagitannins). It has antifungal, antiviral, anticarcinogenic, anti‐inflammatory, antioxidant and antidiabetic properties (Shahrzad, Hodgson, & Narumi, 2001). Caffeic acid has also been reported to increase glucose uptake in rat myocytes (Cheng & Liu, 2000). Quercetin is one of the most common flavonoids occurring mainly in glycosidic forms such as rutin (Havsteen, 1983). Rutin exhibits antioxidants, antibacterial, antitumor, anti‐inflammatory, antidiarrheal, antiulcer, anticarcinogenic, antidiabetic, antimyocardial protection, vasodilator, immunomodulator and hepatoprotective activities (Janbaz, Saeed, & Gilani, 2002).

In conclusion, this study revealed that drying methods significantly altered the phytoconstituents, antioxidant capacity, and enzyme inhibitory effect of moringa leaf with freeze‐drying being the most promising method. The observed alterations in these parameters may be attributed to the fact that increase in temperature observed in both sun and oven‐drying processes could have resulted in UV‐ and heat‐induced destruction of some labile phytoconstituents, while the time taken for air drying to be completed could have resulted in microbial degradation of some valuable phytoconstituents. Hence, freeze‐drying appeared to be the best method of preserving the nutraceutical properties of moringa leaf. However, for practical application, the order of preference of the drying methods which ensures adequate retention of phytoconstituents and possibly biological activities of moringa leaf as observed in this study is oven drying < sun drying < air drying < freeze‐drying, in the order of increasing magnitude.

## CONFLICT OF INTEREST

The authors declare that they do not have any conflict of interest.

## ETHICAL STATEMENT

This study was approved by the Institutional Research Ethical Committee, Federal University of Technology, Akure, Nigeria and the handling and use of the animals were in accordance with NIH Guide.
